# Global changes in mortality rates in polytrauma patients admitted to the ICU—a systematic review

**DOI:** 10.1186/s13017-020-00330-3

**Published:** 2020-09-30

**Authors:** Johanna M. M. van Breugel, Menco J. S. Niemeyer, Roderick M. Houwert, Rolf H. H. Groenwold, Luke P. H. Leenen, Karlijn J. P. van Wessem

**Affiliations:** 1 Department of Trauma Surgery, University Medical Center Utrecht, Heidelberglaan 100, 3585 GA Utrecht, The Netherlands; 2 Department of Clinical Epidemiology, Leiden University Medical Center, Albinusdreef 2, 2333 ZA Leiden, The Netherlands

**Keywords:** Trauma care, Polytrauma patients, Intensive care unit, Mortality

## Abstract

**Background:**

Many factors of trauma care have changed in the last decades. This review investigated the effect of these changes on global all-cause and cause-specific mortality in polytrauma patients admitted to the intensive care unit (ICU). Moreover, changes in trauma mechanism over time and differences between continents were analyzed.

**Main body:**

A systematic review of literature on all-cause mortality in polytrauma patients admitted to ICU was conducted. All-cause and cause-specific mortality rates were extracted as well as trauma mechanism of each patient. Poisson regression analysis was used to model time trends in all-cause and cause-specific mortality.

Thirty studies, which reported mortality rates for 82,272 patients, were included and showed a decrease of 1.8% (95% CI 1.6–2.0%) in all-cause mortality per year since 1966. The relative contribution of brain injury-related death has increased over the years, whereas the relative contribution of death due to multiple organ dysfunction syndrome (MODS), acute respiratory distress syndrome, and sepsis decreased. MODS was the most common cause of death in North America, and brain-related death was the most common in Asia, South America, and Europe. Penetrating trauma was most often reported in North America and Asia.

**Conclusions:**

All-cause mortality in polytrauma patients admitted to the ICU has decreased over the last decades. A shift from MODS to brain-related death was observed. Geographical differences in cause-specific mortality were present, which may provide region-specific learning possibilities resulting in improvement of global trauma care.

## Background

Trauma is the leading cause of death and disability worldwide. Over five million people worldwide are killed annually due to injury resulting from traffic accidents, falls, drowning, burns, poisoning, (self-inflicted) violence, or acts of war. These deaths account for 9% of global mortality—more than that of HIV/AIDS, malaria, and tuberculosis combined. For each death, there are many more hospitalizations, emergency department visits, and doctor’s appointments [1, 2]. Despite many improvements in primary, secondary, and tertiary prevention, e.g., legislation, introduction of computed tomography (CT), and development of advanced trauma life support (ATLS), these numbers show that still there are many trauma victims, and more preventative, diagnostic, and therapeutic options are necessary to reduce these numbers [3].

Several studies have shown that many trauma patients die at a very early stage, either on-site or within the first 48 h after admission [4–6]. However, improvements in injury prevention and trauma care may have caused a right-shift in time of mortality after injury suggesting there has been a shift from a trimodal to a bimodal distribution [3, 6]. Longer survival implicates more intensive care unit (ICU) admissions, rendering improvements in ICU care essential. However, an overview article providing insight in mortality rates of global trauma care is lacking. Such an article could offer important insights in aspects that require further improvement of care as well as research.

The main objective of this systematic review was to assess whether there has been a change in all-cause mortality in polytrauma patients admitted to the ICU. This research’s aim was subdivided in assessing (1) changes in the specific causes of death and (2) differences between geographical locations. A second objective was to assess whether there have been changes in trauma mechanism worldwide.

## Methods

### Search and selection

A systematic review of all published literature according to the Preferred Reporting Items for Systematic Reviews and Meta-Analyses (PRISMA) guidelines was conducted [7]. We aimed to identify all studies that reported on mortality in polytrauma patients (injury severity score (ISS) > 15) admitted to the ICU. On the 26th of February 2020, we systematically searched the PubMed, Cochrane library, and Embase databases. The search terms “polytrauma”, “ICU”, and “mortality” plus their plural forms and synonyms were used. The complete search strings are provided in Appendix 1. Duplicates were removed using an online screening program (Rayyan [8]), and all remaining articles were independently screened by JvB and MN based on the title and abstract. Potentially relevant papers were selected, and full texts were obtained. When correspondence details of the authors were available, they were contacted in case the full text could not be obtained online or from our university library. Articles were excluded when no full text was available; when title, abstract, or full text was not in English, German, French, Spanish, or Dutch; when only a specific subset of trauma patients was researched, e.g., solely severe thoracic trauma; when all included patients suffered from a specific condition e.g., sepsis; and when it concerned reviews or conference abstracts. The references of included papers were screened using the same criteria, as well as the references of relevant and related reviews.

### Quality assessment

Elements from the Critical Appraisal Skills Programme (CAPS), the Methodological Index for Non-Randomized Studies instrument (MINORS), and the Risk of Bias in Non-Randomized Studies of Interventions (ROBINS-I) tool were used to assess the methodological quality of eligible articles [9–11]. Elements included in this assessment comprised clarity and relevance of the study aim, study design, and different types of bias, e.g., selection, detection, and reporting bias. An example of selection bias is when patients were not consecutively included. An example of reporting bias is when the authors did not describe clearly how the cause of death was determined. Suppressing or revealing information selectively is an example of reporting bias. Similar to the MINORS instrument, a score of 0, 1, or 2 points was awarded for each criterion: 0 points were assigned when an item was not reported, 1 point when an item was reported but inadequately, and 2 points when an item was adequately reported, leading to a maximum of 12 points per study.

### Outcome

Data on all-cause mortality, cause-specific mortality, mechanism of injury, and geographical location were extracted from the included articles. The end of data collection from each included study was used (instead of the year of publication) for all analyses to place the data in the right time frame. Cause-specific mortality was stratified in brain injury, thoracic injury, abdominal injury, death by exsanguination, multiple organ dysfunction syndrome (MODS, definitions used by the included articles are shown in Table 1), acute respiratory distress syndrome (ARDS, Table 1), sepsis (Table 1), and death from a cardiac cause. Other causes were categorized as “miscellaneous”. Mechanism of injury was stratified in blunt and penetrating trauma. A second, more detailed analysis for trauma mechanism was performed by using the following categories: traffic accidents, falls from height, workplace accidents, suicide, assault, penetrating injury, and miscellaneous.

**Table 1:** Definition of multiple organ dysfunction syndrome (MODS), acute respiratory distress syndrome (ARDS), and sepsis per included article

Author	MODS	ARDS	Sepsis
Lauwers et al. [12]	≥ 3 failing organs in a sequential pattern	As reported by Petty TL, Fowler AA (1982) Another look at ARDS. Chest 82:98 [61]	Leukocytosis, sustained fever (> 38.5 °C) and identification of a focus of infection either with systemic impact or positive blood cultures
Regel et al. [13]	As reported by Goris RJA, Nuytink HKS, Redl H: Scoring systems and predictors of ARDS and MOF. [62]	Goris RJA, Nuytink HKS, Redl H: Scoring systems and predictors of ARDS and MOF. [62]	Not mentioned
Aufmkolk et al. [14]	≥ 3 failing organs for ≥ 3 sequential days	Not defined	Positive blood culture + ≥ 2 of the following: 36 < Temp. > 38; 4000 < leukocytes > 12,000 or left shift > 10%; heart rate > 90/min; respiratory rate > 20/min or pCO2 < 32 mmHg
Dereeper et al. [15]	Acute renal failure was defined as a blood urea nitrogen (BUN) > 40 and/or creatinine > 2 mg/dl; hepatic failure by a bilirubin > 2 mg/dl or transaminases > 80 IU/l; coagulation abnormalities by a platelet count < 100,000/mm^3^ with either a prothrombin time < 60% of the normal value or an activated partial thromboplastin time > 80 s.	Acute respiratory failure by a PaO2/FiO2 ratio < 250 mmHg or requirement for mechanical ventilation for > 24 h for a respiratory problem	Not mentioned
Nast-Kolb et al. [17]	≥ 2 failing organs for ≥ 3 days (central nervous system not included)	European-American Consensus Conference on ARDS	A source of infection (positive blood culture) plus two or more of the following parameters: temperature 36 (°C) or 38; leukocytes 4000 (nL) or 12,000 or immature neutrophils 10%; heart rate > 90 (beats/min); and respiratory rate > 20 (breaths/min) or pCO2 > 32 mm Hg
Hadfield et al. [16]	Not defined	Not defined	Not defined
Ruiz et al. [41]	Not defined	Not defined	Sequential organ failure assessment (SOFA) score
Ciesla et al. [19]	Denver MOF scoring system	Not defined	Not defined
Zhang et al. [20]	Not defined	Not defined	Not defined
Di Saverio et al. [22]	Not defined	Not mentioned	Not defined
Chen et al. [21]	Not defined	Not defined	Not defined
Dehne et al. [34]	Not defined	Not mentioned	Not mentioned
Van Wessem and Leenen [23]	Denver multiple organ failure (MOF) scoring system	Berlin criteria	Not mentioned
Van Wessem and Leenen [32]	Denver MOFscoring system	Berlin criteria	Not defined

“Not mentioned” means that this particular condition is not included in the article’s analyses. “Not defined” means that this condition is used, but its exact definition is not described

### Statistical analysis

Trends in all-cause mortality and cause-specific mortality over time were visualized and analyzed using Poisson regression models (R Core Team (2015). R: a language and environment for statistical computing. R Foundation for Statistical Computing, Vienna, Austria. URL https://www.R-project.org/). A *p* value < 0.05 was considered statistically significant.

## Results

### Search

**Fig. 1:**
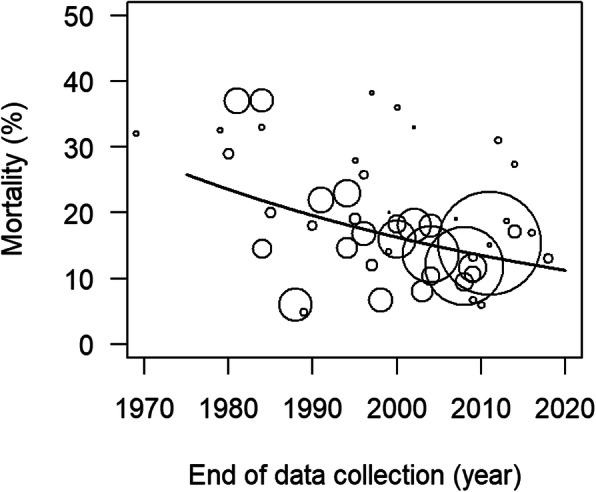
PRISMA flowchart of search, screening, and inclusion strategy

The initial search identified 2704 articles (Fig. 1). One additional article was obtained through personal knowledge of one of the authors. We excluded 429 duplicates after which 2276 articles remained. These were screened by JvB and MN based on their title and abstract. Full texts were then obtained whenever possible. In total, 30 articles were found eligible for this review [12–32]. All references and citations from these 30 articles were screened and identified in Web of Science. However, this did not result in additional relevant articles.

### Characteristics of the studies and study populations

An overview of the study characteristics is provided in Table 2. Thirty studies published between 1985 and 2018 were included in this review with a total of 82,272 patients. Inclusion criteria varied per study and ranged from multiple criteria, such as a minimum ISS or age, to no additional criteria apart from “polytrauma patients” and “admission to the ICU.” Also, the number of included patients varied widely from 20 to 31,154 patients, as well as the study duration with a minimum of 1.5–2 years in the study of Dereeper et al. [15] and a maximum of 30 years for the study of Probst et al. [28]. Most studies were carried out in Europe, and especially Germany was well represented ([12–14, 17, 28, 29, 31, 33–37] 12 out of 19 European studies [12–17, 22, 23, 25, 26, 28, 29, 31–36, 38] were German). Four studies took place in Asia [18, 20, 21, 37], three in the USA [19, 27, 39], two in South America [40, 41], and two in Australia [24, 30].

**Table 2:** Characteristics of studies included in a review of mortality in polytrauma patients admitted to the ICU

Author and year of publication	Study population	Number of included patients	Location	Study duration	Mortality-related outcome
Lauwers et al. (1986) [12]	Blunt trauma, alive ≥ 1 h after ICU-admission, ISS > 25	130	Antwerp, Belgium	Jan 1982 –Feb 1984	Percentages
Hervé et al. (1987) [25]	All	167	Créteil, France	1969 and 1979	Percentages
Kivioja (1989) [26]	All	1169	Helsinki, Finland	1966–1984	Percentages
Goins et al. (1991) [27]	All	2911	Baltimore, USA	July 1985–June 1988	Absolute numbers
Regel et al. (1995) [13]	ISS > 20, ≥ 3 injuries	3406	Hannover, Germany	1972–1991	Percentages, per decade
Regel et al. (1996) [36]	ISS > 20, age 15–65,		Hannover, Germany	1986–1995	Percentages
		342			
Aufmkolk et al. (1997) [14]	ISS ≥ 18, divided in ≥ 65 and < 65 years, >16 years	1154	Essen, Germany	1975–1994	Percentages per age group
Dereeper et al. (1997) [15]	All, children/adults reported separately	97	Brussels, Belgium	1994–1995	Absolute numbers
Pape et al. (1999) [43]	Multiple blunt trauma, ISS > 20, no referrals	61	Hannover, Germany	Oct 1994–Apr 1997	Absolute numbers and percentage
Rixen et al. (2000) [39]	> 16 years, ISS > 16, ICU with cardiorespiratory monitoring	80	New Jersey, USA	N/A	Percentages
Nast-Kolb et al. (2001) [17]	ISS ≥ 16, alive ≥ 24 h after admission	1361	Essen, Germany	1975–1999	Percentages from total population, per 5 years
Hadfield et al. (2001) [16]	All	101	Bristol, UK	1996–1998	Absolute numbers, partly also percentages
Stiletto et al. (2001) [38]	ISS > 15, CCO-measurement	20	Marburg, Germany	1997–1999	Percentages
Ruiz et al. (2013) [41]	Polytraumatized and severely traumatized older than 18 years	72	Puente alto, Chili	2011	Absolute numbers and percentages
Ruscelli et al. (2014) [42]	ISS > 15, ICU admission, death in emergency ward,	747	Cesena, Italy	2007–2009	Absolute numbers and percentages
Ciesla et al. (2005) [19]	ISS > 15, alive > 48 h after trauma, > 15 years	1344	Denver, USA	May 1992–Dec 2003	Absolute numbers and percentages
Dresing et al. (2007) [56]	Age ≥ 18 years, ISS > 15	30	Goettingen, Germany	N/A	Absolute numbers and percentages
Probst et al. (2009) [28]	Blunt trauma	4849	Hannover, Germany	1975–2004	Percentages
Wafaisade et al. (2011) [29]	No missing data, no mild injury	29829	Cologne, Germany	2093–2008	Percentages
Zhang (2011) [20]	All	163	Congqing, China	2006–2009	Absolute numbers
Dewar et al. (2013) [30]	ISS > 15, age > 18 years, AIS < 3, survival > 48 h, no nonmechanical traumas	140	Newcastle, Australia	Dec 2005–Dec 2010	Absolute numbers and percentages
Di Saverio et al. (2014) [22]	ISS > 16	2935	Bologna, Italy	1996–2010	Percentages
Chen et al. (2014) [21]	All	80	Hangzhou, China	Jan 2009–Jun 2013	Absolute numbers
Fröhlich et al. (2014) [31]	ISS > 15, complete data for MOF	31154	Cologne, Germany	2002–2011	Percentages
Dehne et al. (2014) [34]	“Polytraumatized patients”	30	Giessen, Germany	N/A	Absolute numbers
Freitas and Franzon (2015) [40]	“Multiple trauma patients”	117	Sao José, Brazil	Apr 2013–Jul 2014	Absolute numbers
Mazandarani et al. (2016) [37]	Multiple trauma, age > 14 years mortality > 4 h on arrival in ICU.	152	Tehran, Iran	2011–2012	Absolute numbers
Brilej et al. (2017) [33]	ISS > 17, injuries to single region AIS 5, injuries to a single region and abnormal vital signs.	493	Berlin, Germany	2006–2014	Percentages
Van Wessem and Leenen (2018) [23]	Age ≥ 15 years, ISS > 15, no asphyxiation, burns, drowning, and isolated TBI	157	Utrecht, The Netherlands	Nov 2013–Nov 2016	Percentages
Van Wessem and Leenen (2018) [32]	Age ≥ 15 years, ISS > 15, survival > 48 h, no asphyxiation, burns, drowning, and isolated TBI	241	Utrecht, The Netherlands	Nov 2013–April 2018	Absolute numbers and percentages

*ISS* Injury Severity Score, *ICU* intensive care unit, *TBI* traumatic brain injury, *AIS* abbreviated injury scale, *MOF* multiple organ failure, *N/A* not announced

### Quality of the included articles

An overview of the quality of the included studies is given in Table 3. All studies apart from one [21] clearly described their study design. Most studies were retrospective, although data was sometimes collected prospectively in a trauma registry. There was no indication for selection bias in all 30 studies except for three [33, 34, 37]. Either patients were not consecutively included or the inclusion process was not clearly described in these articles. Five studies did not clearly describe how they obtained data on the cause of death [12, 13, 20, 21, 25]. Eleven studies scored the maximum number of points [19, 22, 23, 27, 29–32, 41–43]. The lowest score was five points [37].

**Table 3:** Quality assessment of studies included in review of mortality in polytrauma patients admitted to the ICU

Author	Clearly stated aim	Consecutive patients	Prospective data collection	Selection bias	Detection bias	Reporting bias	Total score
Lauwers et al. [12]	2	2	0	2	1^a^	2	9
Hervé et al. [25]	1	2	0	2	1	2	8
Kivioja [26]	2	2	1^b^	2	2	2	11
Goins et al. [27]	2	2	2	2	2	2	12
Regel et al. [13]	2	2	2^d^	2	1^a^	2	11
Regel et al. [36]	2	1	1	2	2	1	9
Aufmkolk et al. [14]	2	2	0	2	2	2	10
Dereeper et al. [15]	2	2	0	2	2	2	10
Hadfield et al. [16]	2	2	0	2	2	2	10
Pape et al. [43]	2	2	2	2	2	2	12
Rixen et al. [39]	2	1	2	2	2	2	11
Nast-Kolb et al. [17]	2	2	1^b^	2	2	2	11
Stiletto et al. [38]	2	2	2	1	2	2	11
Ruiz et al. [41]	2	2	2	2	2	2	12
Ruscelli et al. [42]	2	2	2	2	2	2	12
Ciesla et al. [19]	2	2	2	2	2	2	12
Dresing et al. [56]	2	2	2	2	1	2	11
Probst et al. [28]	2	2	1^b^	2	2	2	11
Wafaisade et al. [29]	2	2	2^d^	2	2	2	12
Zhang et al. [20]	2	2	0	2	1^a^	2	9
Dewar et al. [30]	2	2	2	2	2	2	12
Chen et al. [21]	2	2	0^c^	2	1^a^	2	9
Di Saverio et al. [22]	2	2	2^d^	2	2	2	12
Fröhlich et al. [31]	2	2	2^d^	2	2	2	12
Dehne et al. [34]	2	0	0	0	2	2	6
Freitas and Franzon [40]	2	2	0	1	2	2	9
Mazandarani et al. [37]	2	2	2	0	2	2	10
Brilej et al. [33]	2	2	2	2	2	2	12
Van Wessem and Leenen et al. [23]	2	2	2	2	2	2	12
Van Wessem and Leenen [32]	2	2	2	2	2	2	12

^a^The authors did not describe how data on mortality was gathered

^b^Part of the data was collected prospectively

^c^Study design not described

^d^Data were collected prospectively; study design was retrospective

### All-cause mortality

All 30 papers reported all-cause mortality rates in their study population (Table 1). Seven studies stratified their total study duration in smaller time spans and reported mortality rates for each time span [13, 17, 22, 25, 28, 29, 31]. All available information was included in our analysis of all-cause mortality.

**Fig. 2:**
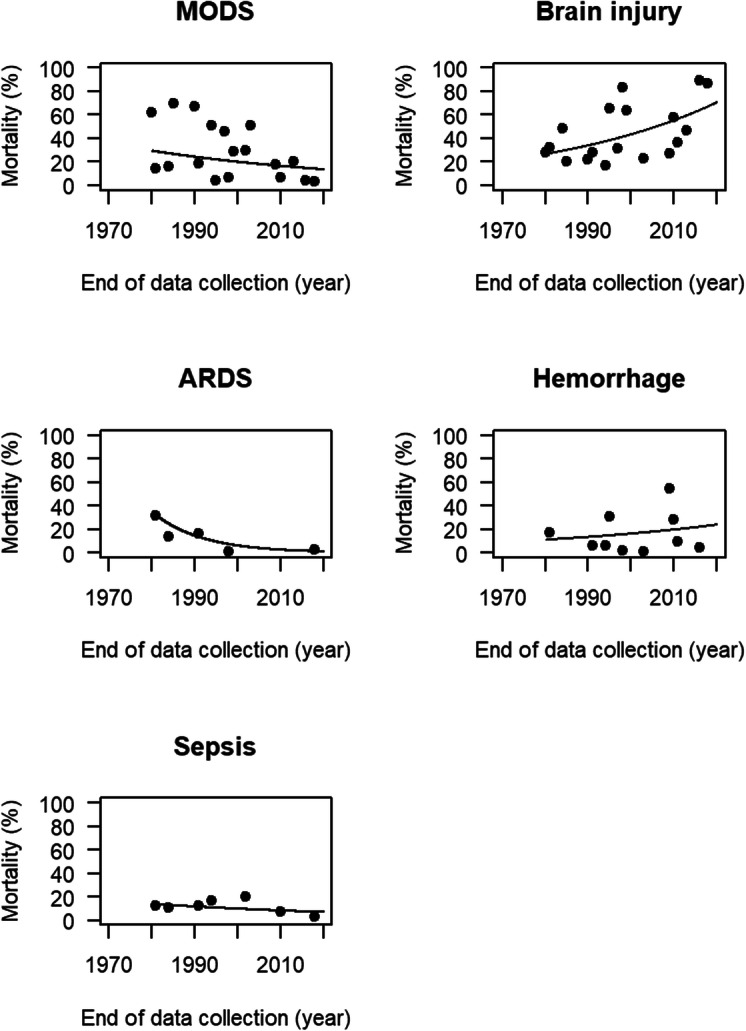
Changes in all-cause mortality in polytrauma patients admitted to the ICU since 1966. Each study is represented by a circle, of which the size is proportional to the number of subjects in the study

All-cause mortality rates in polytrauma patients admitted to the ICU was observed to decrease over time (Fig. 2). We note that there was substantial variation between studies. For example, the study by Mazandarani et al. [37] showed a relatively high mortality (31%) considering its time period (2012), whereas Goins et al. reported a relatively low mortality (6%) for its time period (1988) [27]. Mortality decreased with approximately 1.8% per year (95% confidence interval (CI) 1.6–2.0%, *p* < 0.001).

### Changes in cause of death over time

**Fig. 3:**
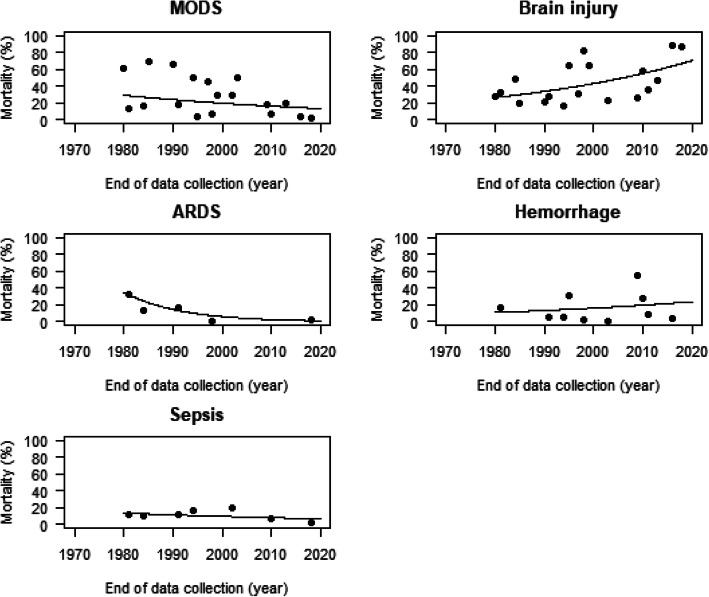
Relative contribution of cause-specific mortality to all-cause mortality in polytrauma patients admitted to the ICU since 1966. Different panels show the relative contribution of different causes of death

Fifteen of the included articles reported data on the individual causes of death [12–17, 19–23, 32, 34, 36, 41]. One paper provided date from five independent time periods leading to a total of 19 data points [17]. Multiple organ dysfunction syndrome (MODS) was reported as the main cause of death in several studies until the end of the last century [13, 14, 17]. At the turn of the century, this altered and brain injury often became the leading cause of death. Figure 3 shows the relative contributions (cause-specific mortality as a percentage of all ICU mortality in trauma patients admitted to the ICU). The relative contribution to ICU mortality of MODS, ARDS, and sepsis decreased over time: relative decreases per year of 1.9% (95% CI 1.2–2.7%), 8.4% (95% CI 6.0–10.6%), and 1.7% (95% CI 0.5–2.9%), respectively. In conjunction with this decrease, an increase was observed for the relative contribution to ICU mortality of brain injury and hemorrhage: relative increases per year of 2.5% (95% CI 1.9–3.0%) and 1.9% (95% CI 1.0–2.9%), respectively.

### Changes in trauma mechanism over time

**Fig. 4:**
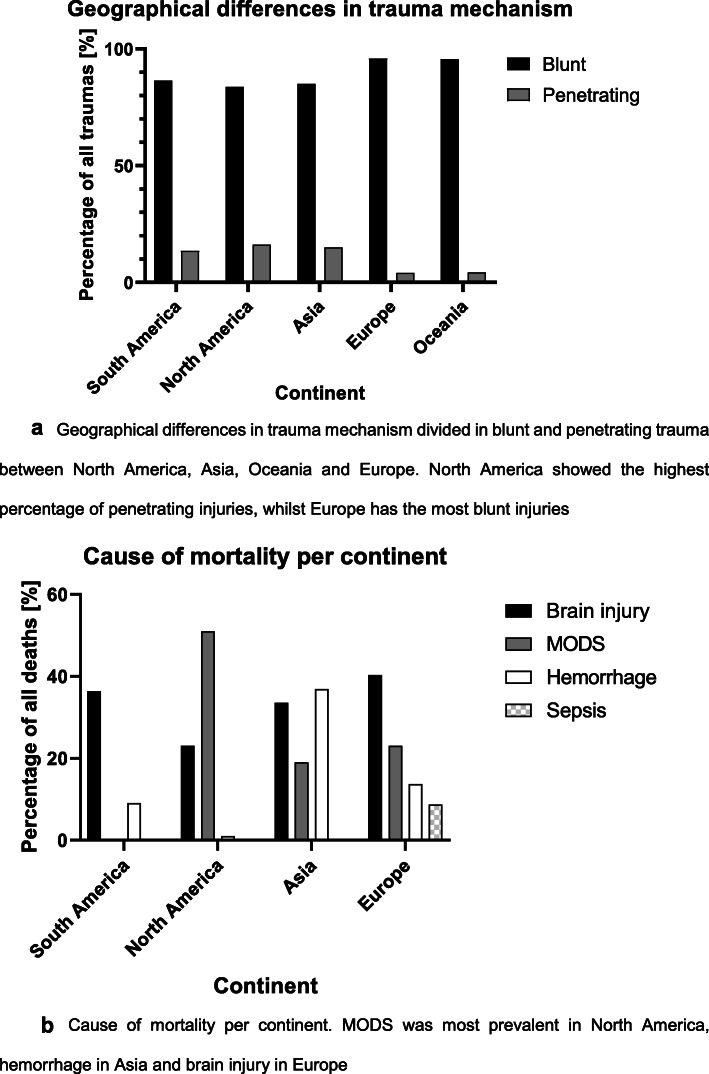
**a** Geographical differences in trauma mechanism divided in blunt and penetrating trauma between North America, Asia, Oceania, and Europe. North America showed the highest percentage of penetrating injuries, while Europe has the most blunt injuries. **b** Cause of mortality per continent. MODS was most prevalent in North America, hemorrhage in Asia, and brain injury in Europe

Thirteen of the 30 included articles provided data on mechanism of injury sustained by their study population [12, 14–16, 20–22, 26, 27, 36, 37, 39, 40]. There have been no changes in the ratio of blunt and penetrating trauma over time (Fig. 4a). Traffic accidents were the most prevalent trauma mechanism reported in all articles, although there has been a decrease of approximately 25% in almost 25 years (Fig. 4b). Falls and workplace accidents were often reported as the second most common trauma mechanism. Assault, suicide attempts, accidental injuries, and penetrating injuries (stab wounds as well as gunshot wounds) were all less common causes of trauma, but were similarly prevalent throughout all years.

### Geographical differences in trauma mechanism and causes of death

The included articles were divided into subgroups representing the continent of the study population. First, differences in all-cause mortality between continents were analyzed for studies published after 2000 to reduce temporal influences (Table 1, Europe *n* = 17, South America *n* = 2, North America *n* = 2, Asia *n* = 3) (Fig. 4). Weighted averages based on the study population of each study resulted in 14.4% all-cause mortality for Europe, 22.6% for South America, 9.6% for North America, and 18.5% for Asia. Further analyses on geographical differences, such as blunt/penetrating trauma and the most prevalent cause of death, can be found in Appendix 2.

## Discussion

This systematic review of all-cause mortality in polytrauma patients admitted to the ICU showed that over the last 35 years all-cause mortality decreased by approximately 1.8% per year. Analysis of cause-specific mortality suggests that this is mainly attributable to decreases in MODS-related and ARDS-related mortality. Mortality due to brain injury on the other hand increased. These observed relative increases and decreases should be seen in relation to each other as all-cause mortality decreased substantially. More specifically, before the turn of the century, organ failure was a more prevalent cause of death than brain injury. These findings are in line with the results from the study by Trunkey [5]. He suggested a trimodal distribution of immediate, early, and late deaths following trauma with the late deaths occurring several days to weeks after the initial injury. The cause of death in this phase was most commonly due to sepsis and MODS. However, since this classification system is over 30 years old, this trimodal distribution does not reflect the current situation anymore, as shown in previous studies [6].

Probably many factors, both pre-hospital and in-hospital, have contributed to the decrease in mortality throughout the past decades. Preventive strategies and legislation, such as obligatory use of seatbelts, as well as advances in diagnostic tools, resuscitation protocols, and peri-operative and surgical procedures have played an important role [2, 3, 44–47]. In addition, many improvements in trauma care regarding prevention and treatment of MODS/ARDS/sepsis have potentially led to the observed shift towards a larger relative contribution of brain injury-related death. According to Nast-Kolb et al. MODS-related mortality decreased due to an improved overall performance of trauma management and ICU care [17]. They identified several specific, major changes such as volume resuscitation, mechanical ventilation with airway pressure limitation, damage control treatment and early enteral nutrition as being of most importance.

High brain injury-related mortality could partially be explained by the trauma mechanism. Brain injury is often a consequence of blunt injury. It can be divided in primary brain injury, resulting in direct neuronal damage from the accident, and in secondary injury occurring at a later stage due to hypoxemia, hypotension, seizures, and intra-cranial hypertension. Secondary injury is a major contributor to mortality [16, 48]. According to Hadfield et al., secondary insults are preventable and treatable, and the main aim of critical care must be to prevent such secondary insults [16]. However, hypoxemia and hypotension may remain important causes of mortality, as they are complications of massive hemorrhage, which often used to be fatal at the site of the accident or in the emergency department, but is not anymore.

Interestingly, hemorrhage-related death in the ICU increased over time. This is in contrast with a large review showing that exsanguination-related death decreased over time (approximately 20% in 20 years) when the entire trajectory from the pre-hospital phase until the ICU was observed [3]. The authors suggested that the improvements in hemorrhage management and implementation of ATLS decreased mortality within 60 min after admission [49, 50]. Also, rapid diagnostics with 24-h access to an onsite CT scanner and the introduction of the damage-control approach further reduced the probability of exsanguination soon after hospital admission [51–53]. Before the introduction of damage control resuscitation about 20 years ago, surgeons would operate and perform definitive interventions. This often led to metabolic derangement and/or death as severely injured patients frequently do not have the physiological reserve to undergo definitive surgery. On the other hand, nowadays, patients may survive the initial phase of trauma care in the ED and operating theater but may bleed out in the ICU due to new onset or uncontrolled surgical bleeding. Our findings suggest that there is a relative increase (in relation to a decrease in other causes of mortality) in exsanguination once admitted to the ICU.

One of the studies included in this review studied changes in ICU mortality from exsanguination over a 15-year period and found no changes [22]. Considering these findings, it may be worthwhile to focus on preventative and therapeutic options for exsanguination in the ICU setting.

Analysis of the data per continent showed slight differences in all-cause mortality. Alternatively, penetrating injuries were most common in North America which has been previously reported [16, 54]. We recommend more research on differences between continents and countries and the influence of different trauma systems on these variances to create learning opportunities and improvements in global trauma care.

This review has several limitations. First, the term “polytrauma”, one of our inclusion criteria, has always been a topic of debate in literature [35, 55]. Several of our included studies used different definitions, e.g. ISS > 15 by Fröhlich et al. [31] and ISS > 25 by Lauwers et al. [12] (Table 1). A start at gaining consensus was made with the international meeting in 2012 [35]. Further work should build upon this meeting and should focus on estimating the risk of mortality and predicting the requirement of therapeutic care on an individual basis. This will help to apply new findings to the right patients and in comparing study results more accurately. Similarly, the terms MODS, ARDS and sepsis were defined differently in different studies (Appendix 2). Also, whereas some studies looked at all deaths during ICU admission, others included solely data from a pre-defined period, such as 30-day ICU mortality. Yet, study outcomes were compared in this study as these contain the best data currently available. Also on other levels the included study populations were similar, but not entirely equal, e.g., Lauwers et al. [12] only included patients suffering from blunt trauma, while other authors also included penetrating trauma [13, 15, 20].

A second limitation is the extensive period covered in this review with a relatively small number of included articles. We suspect that more studies reported all-cause mortality, but if these numbers were not reported in the title or abstract, these articles were not identified by our search (Fig. 1). This large time span might have introduced bias, e.g., due to changes in causes of death that were of interest in the concerning time period or due to the development of establishing specialized trauma centers, e.g., for neurotrauma. Another limitation concerns a substantial number of included articles reported on data from the German trauma registry, which limits the generalizability of our findings. Also, in this review work-related injury is reported as a trauma mechanism—as do many article references in our review—although strictly it is not a trauma mechanism by itself. Rather, it is an umbrella term for trauma mechanisms, such as fall from height, falling objects, crushing injuries, and machinery injuries. A final limitation is that several studies provided data based on relatively long study periods of 10, 15, and 20 years [14, 19, 22]. Since studies were analyzed based on the end of data collection, rather than the years from which information was obtained, this could have influenced the results of this review.

## Conclusion

In conclusion, in this review of polytrauma patients admitted to the ICU, the all-cause mortality decreased over the last decades. This decline could be considered a success of the improvements in trauma care. Before the turn of the century MODS was the leading cause of death, whereas nowadays it is brain injury.

## Data Availability

The datasets used and/or analyzed during the current study are available from the corresponding author on reasonable request.

## Appendix 1. Search strings

PubMed

((“Multiple Trauma”[Mesh] OR polytrauma [tiab] OR “multiple trauma” [tiab] OR “massive trauma” [tiab] OR “multiple injury” [tiab] OR “polytraumatized patient*” [tiab] OR “multiple trauma” [tiab] OR “severely injur*” [tiab]) AND (“Intensive Care Units”[Majr] OR ICU* [tiab] OR “Intensive Care Unit*” [tiab] OR “intensive care department” [tiab] OR “intensive treatment unit*” [tiab]) AND (“Mortality”[Mesh] OR mortality [tiab] OR death [tiab] OR casualt* [tiab] OR outcome* [tiab]))

Embase

(‘multiple trauma’/exp OR ‘massive trauma’:ti,ab,kw OR ‘multiple injury’:ti,ab,kw OR polytrauma:ti,ab,kw OR ‘polytraumatized patient’:ti,ab,kw OR ‘multiple trauma’:ti,ab,kw) AND (‘intensive care unit’/exp OR icu:ti,ab,kw OR icus:ti,ab,kw OR ‘intensive care department’:ti,ab,kw OR ‘intensive care units’:ti,ab,kw OR ‘intensive treatment unit’:ti,ab,kw OR ‘intensive care’:ti,ab,kw OR ‘special care unit’:ti,ab,kw) AND (‘mortality’/exp OR death:ti,ab,kw)

## Appendix 2. Further analyses on geographical differences

Differences in the ratio between penetrating and blunt trauma were analyzed. Summary statistics (percentages) are presented here. Because of the limited amount of data, no comparative statistical tests were performed. Seventeen articles included both trauma types in their study population and reported complete data (North America (USA): *n* = 3 [19, 27, 39], South America: *n* = 2 [40, 41], Asia: *n* = 2 [20, 21], Oceania (Australia): *n* = 2 [24, 30], Europe: *n* = 8 [15, 16, 22, 23, 29, 31–33]). Weighted averages per continent were calculated based on patient numbers. In all five continents, the majority of the traumas were blunt (Fig. 4a). North America had the highest percentage of penetrating trauma (16%), followed by Asia (15%), South America (13%), and then Europe and Oceania (both 4%).

Further differences in the causes of mortality per continent were studied. The four most common causes were selected: brain injury, MODS, hemorrhage, and sepsis. Fourteen articles reported complete data and were included in this analysis (North America (USA): *n* = 1 [19], South America: *n* = 1 [41], Asia: *n* = 2 [20, 21], Europe: *n* = 10 [12–17, 22, 23, 32, 34]). Again, weighted averages were calculated. MODS was highest in North America (51% of all deaths), while brain injury was most common in Europe (40%), and hemorrhage was most prevalent in Asia (37%) (Fig. 4b). Over time brain injury-related mortality substantially increased in Europe, whereas MODS-related death decreased substantially. It should be noted that the number of included articles per continent is small and that, as a consequence, there is a substantial risk of bias, e.g., over time the interest in reporting (different) causes of death may have changed and specialized trauma centers may have been established.

## Abbreviations

ARDS: Acute respiratory distress syndrome
ATLS: Advanced trauma and life support
CAPS: Critical Appraisal Skills Programme
CT: Computed tomography
ICU: Intensive care unit
MINORS: Methodological Index for Non-Randomized Studies instrument
MODS: Multiple organ dysfunction syndrome
PRISMA: Preferred Reporting Items for Systematic Reviews and Meta-Analyses
ROBINS-I: Risk of Bias in Non-Randomized Studies of Interventions
